# Oral hygiene and health-related quality of life in institutionalized older people

**DOI:** 10.1007/s41999-021-00547-8

**Published:** 2021-07-27

**Authors:** Riitta K. T. Saarela, Kaija Hiltunen, Hannu Kautiainen, Hanna-Maria Roitto, Päivi Mäntylä, Kaisu H. Pitkälä

**Affiliations:** 1 Department of Social Services and Health Care, City of Helsinki, Oral Health Care, P.O. Box 6009, 00099 Helsinki, Finland; 2 Department of Oral and Maxillofacial Diseases, Faculty of Medicine, University of Helsinki, Helsinki, Finland; 3 Primary Health Care Unit, Kuopio University Hospital, Kuopio, Finland; 4 Folkhälsan Research Center, Helsinki, Finland; 5 Department of Social Services and Health Care, City of Helsinki, Geriatric Outpatient Clinic, Helsinki, Finland; 6 Institute of Dentistry, University of Eastern Finland, Kuopio, Finland; 7 Kuopio University Hospital, Oral and Maxillofacial Diseases, Kuopio, Finland; 8 Department of General Practice and Primary Health Care, University of Helsinki, Helsinki, Finland; 9 Helsinki University Hospital, Unit of Primary Health Care, Helsinki, Finland

**Keywords:** Aged, Institutional care, Oral hygiene, Health-related quality of life

## Abstract

**Purpose:**

We evaluated the level of oral hygiene and its association with oral health status and need for oral treatment among older residents in long-term care facilities. In addition, the association between oral hygiene level and health-related quality of life (HRQoL) was explored.

**Methods:**

This cross-sectional study assessed 231 dentate residents in long-term care facilities (71% female, mean age 81 years, 70% had dementia). Nurses assessed residents and completed questionnaires on participants’ background information, diagnoses, oral healthcare habits, and HRQoL with the 15D instrument. Two qualified dentists performed clinical oral examinations (number of teeth, plaque index, periodontal condition, open caries lesions, and dry mouth). We used a modified plaque index (PI) to measure the level of oral hygiene (good, moderate, and poor) and calculated the clinical Asymptotic Dental Score (ADS) to determine the oral inflammation burden.

**Results:**

Of the residents, 21% had good, 35% moderate, and 44% poor oral hygiene according to PI. Poor oral hygiene was associated with poorer cognitive status (*P* = 0.010) and higher oral inflammation burden (*P* < 0.001). Moreover, poor oral hygiene was associated with poorer HRQoL in a correlation analysis adjusted for age and gender.

**Conclusions:**

Oral hygiene of older individuals in long-term care is insufficient. Poor oral hygiene is a marker for poor HRQoL. Residents also have a high burden of oral inflammatory diseases and a need for dental care. Older residents’ oral hygiene and HRQoL may be improved with oral care education of caregivers and regular dental check-ups.

## Key summary points

**Aim:**

We evaluated oral hygiene level and its association with oral health and general health-related quality of life (HRQoL) among older residents in long-term care facilities.

**Findings:**

Only one-fifth of residents had good oral hygiene. Poor oral hygiene was associated with poor oral health and diminished HRQoL.

**Message:**

Oral hygiene, oral health, and HRQoL may be improved with oral care education of caregivers, professional cooperation, and regular oral healthcare of older residents in long-term care facilities.

## Introduction

Oral health is an important component of health, well-being, and quality of life. Edentulousness has decreased and most older people, also those residing in long-term care facilities, have some of their natural teeth left [1, 2]. However, about two in five residents in long-term care settings are edentulous [2–6]. In assessing oral health, the most common measurements have been number of present teeth and prevalence of oral diseases such as dental caries, periodontitis, and mucosal infections [7]. Of the dentate, 40–77% have caries [3–5], and periodontal disease is very common [4, 6] in institutional settings. The new definition of oral health is multi-faceted and includes the ability to speak, smile, smell, taste, touch, chew, swallow, and convey a range of emotions through facial expressions with confidence and without pain, discomfort, or disease of the craniofacial complex [8]. Oral cleanliness is crucial in maintaining good oral health and the basis for prevention of oral diseases among older residents in long-term care facilities.

Oral hygiene is poor among vulnerable, care-dependent institutionalized older people [4–6, 9], particularly among those residents needing assistance with oral hygiene [10, 11]. This situation could be explained by many staff-related factors such as high workload, lack of time, poor attitudes, and lack of knowledge regarding the importance of oral hygiene in prevention of oral diseases [12, 13]. In addition, some resident-related characteristics, such as self-determination, care-resistant behavior, and low level of cooperation, may be barriers to maintaining daily oral hygiene [3, 14]. There is some evidence that carers’ education may improve oral hygiene among care-dependent nursing home residents [15, 16]. Oral health and oral health-related quality of life (OHRQoL) among institutionalized older people are increasingly being investigated [17]. Previous studies have reported poor OHRQoL among institutionalized older residents due to their generally poor oral health [3, 18]. However, studies exploring the role of oral cleanliness in general health-related quality of life (HRQoL) in this specific population are lacking. The aim of this study was to explore oral hygiene level, oral health status, and need for oral treatment among older residents in long-term care facilities. Another aim was to assess how oral hygiene level is associated with oral diseases and HRQoL.

## Materials and methods

This study is part of a larger project exploring the nutritional status and quality of nutritional care among long-term care residents in Helsinki, Finland. All residents aged 65 years and over who were permanently living (*N* = 3673) in long-term care settings (nursing homes and assisted living facilities) in Helsinki were approached to participate. The participation rate was 68% (*N* = 2482). Registered nurses most familiar to the residents collected the nutrition data in March 2017. They received thorough training in all assessment protocols before data collection. The questionnaire included items on demographic factors, health and functional status, medical history, oral care, and HRQoL.

They also retrieved dementia diagnosis and use of medication from medical records. Nurses used the Mini-Mental State Examination (MMSE) to assess participants’ cognition [19] and the Clinical Dementia Rating (CDR) “Personal care item” [20] to assess dependence on activities of daily living (ADL). Nurses assessed residents’ nutritional status using the Mini Nutritional Assessment (MNA) [21], which is an 18-item tool used to assess nutritional risk (0–30 points). MNA score < 17 indicates malnourishment, a score between 17 and 23.5 indicates risk for malnourishment, and a score > 23.5 indicates good nutritional status.

Residents’ HRQoL was measured using the 15D quality of life instrument [22]. The 15D is a generic, comprehensive, 15-dimension, standardized measure of HRQoL that can be self-administered or proxy-rated. We used it both as a profile and as a single index score measure. It consists of 15 dimensions (mobility, vision, hearing, breathing, sleeping, eating, speech, excretion, usual activities, mental function, discomfort and symptoms, depression, distress, vitality, and sexual activity) with five ordinal levels. The single index score of the 15D instrument ranges from 0 to 1 and represents the overall HRQoL.

Nurses assessed residents’ frequency of tooth brushing with the following question: “Are the resident’s teeth/dentures brushed daily independently or with help?” (yes/no). Similarly, nurses evaluated residents’ swallowing difficulties with a yes/no question. The use of oral healthcare services was assessed with the question: “When was the last oral examination performed by a dentist or a dental hygienist?” The responses were categorized as follows: 1 = less than 1 year ago; 2 = 1–3 years ago; 3 = more than 3 years ago.

### Clinical oral examination

In 2018–2019, two qualified and calibrated dentists performed a comprehensive clinical oral examination on a random sample of 393 residents. Residents gave informed consent also to the oral health study (FINORAL—Finnish Oral Health Study in Long-Term Care). Calibrated dentists carried out a clinical oral examination in long-term care settings with participants lying in their bed or sitting in a chair during the oral examination. Dentists used a mouth mirror and a WHO probe, and they were equipped with loupes (Merident Optergo MO Ultralight Flip-up) and an attached headlamp (Merident Optergo DeLight LED). The clinical oral examination included recording the number of teeth, plaque index, periodontal condition (gingival index, bleeding on probing, periodontal pocket depth measurements), open caries lesions visible to the naked eye, and dryness of the mouth.

Residents were assessed as dentate if she/he had at least one visible tooth. Over half of the examined residents (*N* = 231) were classified as dentate. We divided these dentate participants into three groups according to the amount of dental plaque. We used a modified Silness–Löe [23] plaque index (PI) to measure the level of oral hygiene: 0 = no plaque, 1 = thin plaque layer at the gingival margin, only detectable by scraping with a probe; 2 = moderate layer of plaque along the gingival margin, interdental spaces free, but plaque visible to the naked eye; 3 = abundant plaque along the gingival margin, interdental spaces filled with plaque; 4 = whole tooth crown covered with plaque, and calculated the mean value for each resident. PI < 2 indicates good oral hygiene, 2–2.9 moderate oral hygiene, and ≥ 3 poor oral hygiene.

The dentists also visually assessed whether food debris was present in the subject's mouth. Dentists clinically evaluated signs of dryness in the mouth (modified from Osailan et al. [24]): normal salivation, reduced salivation (mirror sticks to buccal mucosa or tongue, frothy saliva), or dry mouth (glassy appearance of oral palate, lobulated/fissured tongue). The dentists registered open caries lesions visible to the eye. Periodontal condition was measured with gingival index (GI; 0 = no inflammation to 3 = severe gingival inflammation), periodontal pocket depth (PPD) registered as the deepest PPD finding for each tooth (< 4 mm, 4–5 mm, ≥ 6 mm), and bleeding on probing (BOP; yes/no for each tooth) and further calculated as the percentage of BOP-positive teeth. We calculated the oral inflammation burden by means of a clinical Asymptotic Dental Score (ADS) (modified from Janket et al. [25]) (Table 1). Points determining the index number of ADS were given according to the severity of the disease/sign. The sum of the assigned points (0–9) was the final ADS score; ADS 0–2 indicated no or low, 3–4 moderate, and 5–9 high asymptomatic oral inflammation burden.

**Table 1:** Clinical Asymptotic Dental Score (ADS)

Variable	Points
Retained root remnants	0 = no root remnant 1 = one root remnant 2 = ≥ 2 root remnants
Gingivitis	0 = no gingivitis 1 = gingivitis
Deepened periodontal pockets (number of teeth with probing depth, PD, 4–5 mm plus weighted number of teeth with PD ≥ 6 mm	0 = no pockets 1 = 1–3 pockets 2 = 4–10 pockets 3 = ≥ 11 pockets
Dental caries/edentulism	0 = no caries 1 = 1–3 caries lesions 2 = 4–7 caries lesions or one toothless jaw 3 = ≥ 8 caries lesions

**Table 2:** Characteristics of the residents in long-term care facilities divided into groups according to plaque index (PI) and the level of oral hygiene

Characteristic	PI < 2 Good oral hygiene *N* = 48	PI 2– < 3 Moderate oral hygiene *N* = 81	PI ≥ 3 Poor oral hygiene *N = *102	*P* value*
*Nurses’ assessment*				
Age, mean (SD)	81 (8)	81 (9)	81 (9)	0.83
Female, *N* (%)	32 (67)	58 (73)	75 (74)	0.42
Education, < 8 years, *N* (%)	17 (39)	30 (39)	32 (36)	0.71
Dementia, *N* (%)	28 (58)	55 (68)	79 (77)	0.015
MMSE, mean (SD)	16.5 (6.8)	13.9 (7.7)	12.8 (7.0)	0.010
CDR- requires much help with personal care, *N* (%)	35 (80)	71 (91)	93 (91)	0.073
Number of continuous medications, mean (SD)	9.6 (3.4)	9.7 (3.9)	8.0 (3.3)	0.002
MNA, *N* (%)				0.052
≥ 24 (good nutritional status)	11 (25)	17 (23)	11 (12)	
17–23 (risk of malnutrition)	28 (64)	45 (62)	65 (71)	
< 17 (malnutrition)	5 (11)	11 (15)	16 (17)	
Swallowing difficulty, *N* (%)	9 (20)	15 (20)	18 (19)	0.89
Daily toothbrushing, *N* (%)	45 (94)	73 (91)	92 (91)	0.62
Latest oral examination performed by a dentist/dental hygienist less than 1 year ago, *N* (%)	27 (56)	36 (44)	43 (42)	0.13
HRQoL index score, mean (SD)	0.650 (0.148)	0.628 (0.120)	0.613 (0.125)	0.098
*Dentists’ clinical oral examination*				
Number of teeth, mean (SD)	15.4 (7.9)	14.1 (8.3)	12.8 (8.0)	0.058
Brushing teeth independently, *N* (%)	14 (30)	15 (19)	16 (16)	0.069
Food depris, *N* (%)	19 (40)	44 (56)	69 (70)	< 0.001
BOP, %	70 (36)	84 (29)	91 (25)	< 0.001
Caries, * N* (%)	21 (46)	48 (62)	63 (62)	0.092
Reduced salivation, *N* (%)	32 (68)	59 (75)	81 (82)	0.060
ADS, mean (SD)	3.2 (1.7)	4.0 (1.7)	4.5 (1.8)	< 0.001

*SD* standard deviation; *MMSE* Mini Mental State Examination; *CDR* Clinical Dementia Rating Scale; *MNA* Mini Nutritional Assesment; *HRQoL* Health Related Quality of Life; *BOP* Bleeding on probing; *ADS* Clinical Asymptomatic Dental Score

*P for linearity

Datasets of the FINORAL and original Helsinki Nutrition Study were then combined. Thus, we obtained background information, nutritional status, clinical oral examination, and 15D data for 231 dentate residents.

### Statistical analysis

Data are expressed as mean and standard deviation (SD) or counts with percentages. The linearity across the three PI groups was evaluated using the Cochran–Armitage test (Chi-square test for trend), logistic models, and analysis of variance with an appropriate contrast (orthogonal). In case of violation of assumptions (e.g., non-normality), a bootstrap-type test was used. Adjusted correlation coefficients between the PI and dimensions of 15D were calculated by using Spearman's rank correlation method. The normality of variables was evaluated graphically and by using the Shapiro–Wilk W test. The Stata 16.1, StataCorp LP (College Station, TX, USA) statistical package was used for the analysis.

## Results

Residents’ mean age was 81 years, 71% were female, and one-third had a low level of education (< 8 years). Of residents, 70% had diagnosed dementia and 86% needed much assistance in personal care according to the CDR. About 14% of the residents were malnourished. According to nurses’ assessment, 18% had swallowing difficulties (Table 2).

Of the residents, 21% (*N* = 48) had good oral hygiene, whereas 35% (*N* = 81) had moderate and 44% (*N* = 102) poor oral hygiene according to the PI. The level of oral hygiene was significantly associated with dementia (*P* = 0.015) and poorer cognitive status (*P* = 0.010). The mean MMSE score of the residents with good oral hygiene was 16.5. The corresponding figures for the moderate and poor hygiene groups were 13.9 and 12.8, respectively. According to nurses’ assessment, almost all residents (91%) had their teeth brushed daily, although less than one-fifth (19%) brushed their teeth independently. Less than half of the residents had undergone an oral examination within the past year.

In dentists’ examination, the residents had an average of 13.8 (SD 8.1) teeth left. Of the residents, 57% had open caries lesions visible to the naked eye. These oral problems were equally common at all oral hygiene levels. According to dentists’ clinical evaluation, 74% of the residents had reduced salivation. Over half of the subjects (57%) had food debris in their mouth, and it was linearly associated with oral hygiene (*P* < 0.001). In the group of residents with good oral hygiene, the mean prevalence of BOP-positive teeth was 70%. The corresponding figures in the moderate and poor oral hygiene groups were 84% and 91%, respectively (*P* < 0.001). The burden of oral inflammatory diseases in residents according to ADS was associated with the level of oral hygiene (*P* < 0.001). In residents with good oral hygiene, the mean ADS score was 3.2. The corresponding figures in the moderate and poor oral hygiene groups were 4.0 and 4.5, respectively.

**Fig. 1:**
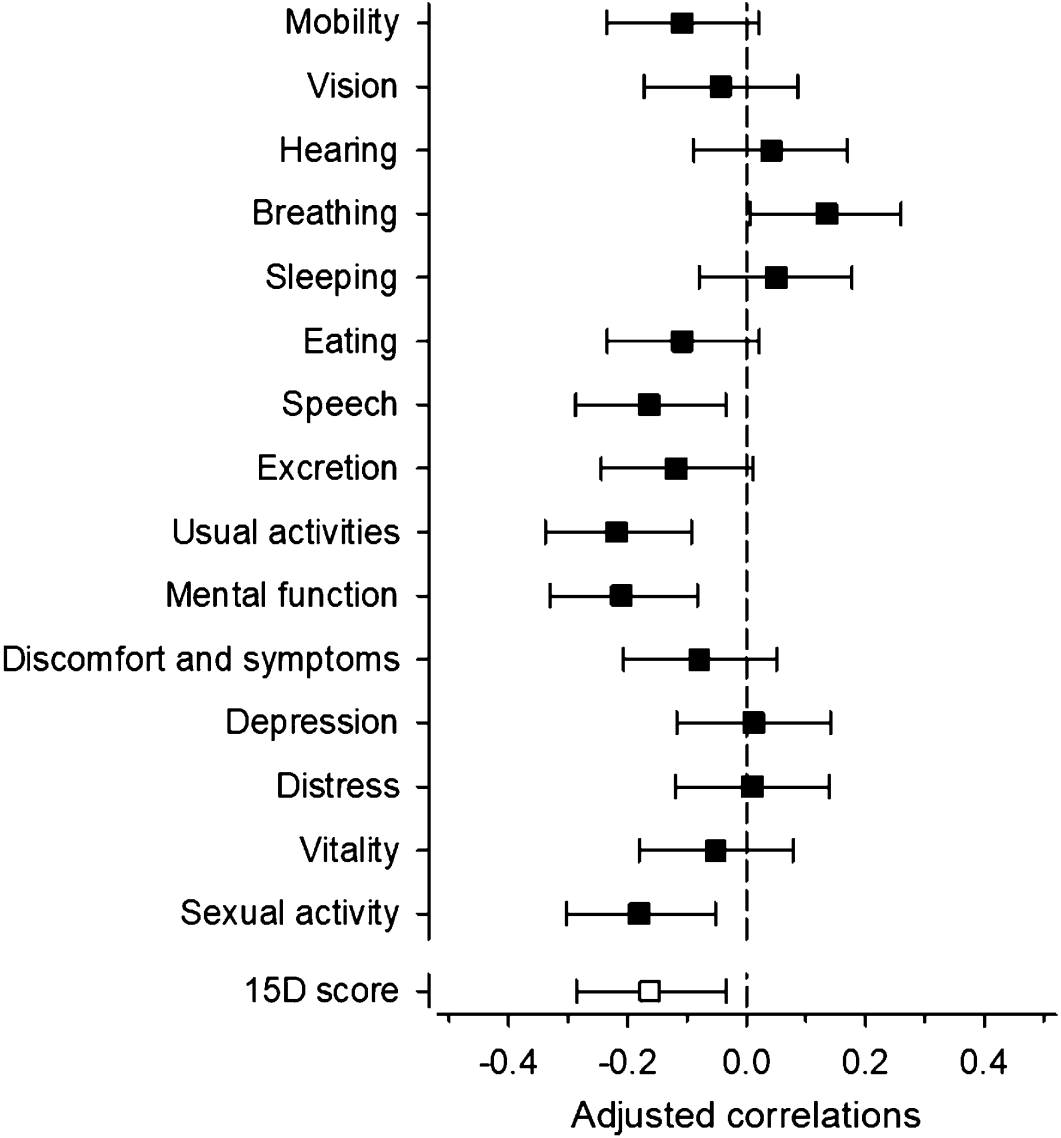
Spearman’s correlations with 95% confidence intervals between plaque index and dimensions of 15D adjusted for age and gender

Total 15D score did not differ between the oral hygiene groups. Figure 1 shows the results from the partial correlation analysis between the PI as a continuous variable and the dimensions of the 15D adjusted for age and gender. Total 15D score was negatively correlated with the plaque index. Of the dimensions, speech, usual activities, mental function, and sexual activity were negatively correlated with plaque index.

## Discussion

Among long-term care residents, only one in five had good oral hygiene according to the plaque index, whereas 35% had moderate and 44% poor oral hygiene. Periodontal level of inflammation measured by BOP seemed poor because most of the teeth (70–91%) had a positive BOP finding in all oral hygiene groups. Oral inflammation burden according to ADS was on average moderate. Of the residents, 57% had open caries lesions, which is one component of ADS. These findings indicate poor oral health and a great need for oral treatment. HRQoL according to 15D score was negatively correlated with the plaque index.

A strength of our study is that two qualified and calibrated dentists performed thorough clinical oral examinations and determined PI to measure the level of oral hygiene. In addition, we used well-validated instruments, such as MNA, MMSE, CDR, and 15D, in data collection and well-trained nurses to collect the data. We also used a structured questionnaire validated in previous nutritional studies in Helsinki in 2003–2011 [26]. To our knowledge, this is the first study to explore general HRQoL and its relationship with oral hygiene in institutional settings. A limitation of our study is its cross-sectional design, which makes it impossible to draw causal relationships between the level of oral hygiene and its associated factors and HRQoL. Another limitation is that HRQoL was evaluated by proxies who may overestimate the impact of cognitive decline on HRQoL compared to participants [27]. However, 15D can be self-reported or evaluated by proxy [22].

In line with earlier studies, poor oral hygiene [4, 6] and oral diseases were common in our study population [17]. More than half of the residents had at least one open caries lesion, consistent with other studies [3–5]. Gingival bleeding is a sign of inflammation of periodontal tissues, and it can reflect gingivitis or periodontitis. In both cases, accumulation of dental plaque is a key contributing factor. In this study, however, deepened periodontal pockets were included in the ADS calculation and were not specifically reported. There is evidence that periodontitis is a risk factor for general health and diseases such as coronary heart disease and atherosclerosis [28]. In accord with earlier research, the prevalence of gingival bleeding measured by BOP was very high [4, 6], and it was associated with the level of oral hygiene. In addition to this, a large burden of oral inflammation measured by ADS was associated with poor oral hygiene. This indicates a great need for oral treatments such as restorative and periodontal care. Furthermore, polypharmacy and its side-effect of reduced salivation as well as swallowing difficulties were highly prevalent in the study population, consistent with earlier reports [5, 29]. Reduced salivation is a risk factor for caries and mucosal infections [3, 30]. In older persons with swallowing difficulties, good oral hygiene is essential to prevent both oral diseases and aspiration pneumonia [31]. Nursing staff must have the knowledge and ability to recognize early signs of residents’ oral problems and diseases, and the possibility to refer residents to oral healthcare services. Thus, there needs to be higher priority and support for those providing oral care [32]. There was a mismatch between expressed oral hygiene measures being declared by staff against the findings of the levels of oral hygiene.

Our findings also suggest that poor oral hygiene of older care-dependent residents in long-term care facilities is correlated with poorer general HRQoL measured by the 15D instrument. Several studies have shown that OHRQoL and oral health problems are associated [3, 18, 33]. However, there is a scarcity of studies exploring the relationship between general HRQoL and oral health in long-term care settings. Furthermore, to our knowledge, no prior studies have investigated the relationship between oral hygiene and HRQoL. Some evidence suggests that receiving proper dental care and rehabilitation may improve HRQoL among long-term care residents [34, 35]. Paying attention to oral hygiene and improving tooth brushing are practical and easy ways to enhance residents’ HRQoL. Further research is needed to confirm these findings.

Good daily oral hygiene of dependent residents is the best way to prevent oral disorders. In our study, nurses reported that the teeth of most residents were brushed at least once a day. Clinical oral examination showed that the level of oral hygiene was poor since about 80% of residents had plaque on the surfaces of teeth. Our finding is in line with previous studies [9, 36, 37]. In addition, nearly 60% of residents had food debris in their mouth. Consistent with recent studies, residents with poor oral hygiene had significantly poorer cognitive status than those with better oral hygiene [7]. Previous studies have reported that care-dependence was significantly associated with inadequate oral hygiene [10, 11]. Our study population comprised old, cognitively impaired, and care-dependent residents, and in most cases the nursing staff performed daily tooth brushing. We did not find a statistically significant difference in the level of oral hygiene according to who performed the daily tooth brushing.

Our results suggest that adequate tooth brushing and oral hygiene are challenging in long-term care residents [38]. Personnel in long-term care facilities need training to enhance understanding of the impact of oral hygiene on oral health, general health, and quality of life of residents. Theoretical knowledge is the basis for improving oral hygiene and oral health in this population, but in itself is not sufficient to make the necessary changes. There is also a need for professional cooperation [39]. Oral healthcare professionals (dental hygienists and dentists) should provide hands‐on guidance regarding oral hygiene procedures and discussions on oral care routines [40]. Nursing personnel also need to overcome barriers such as residents’ responsive behaviors [41]. Dental hygienists can motivate and coach nursing personnel in long-term care facilities to tackle these difficulties [42]. Less than half of the residents had undergone an oral examination in the past year, despite having many risk factors for oral diseases, including impaired cognition, polypharmacy, and dry mouth.

## Conclusion

Oral hygiene of dentate older people in long-term care is inadequate. Poor oral hygiene is associated with diminished HRQoL. Oral hygiene and HRQoL might be improved with oral care education of caregivers. Poor oral hygiene often indicates poor oral health. Thus, residents have a great risk of oral inflammation and a need for care from oral health professionals. Residents should have regular oral health check-ups.
